# *In Vivo* Anti-Malarial Activity of the Aqueous Root Extract of *Euclea divinorum* Hiern (Ebenaceae) against *Plasmodium berghei* ANKA

**DOI:** 10.1155/2022/2640648

**Published:** 2022-07-30

**Authors:** Fentaw Girmaw, Ephrem Engidawork

**Affiliations:** ^1^Department of Pharmacy, College of Medicine and Health Science, Woldia University, Woldia, Ethiopia; ^2^Department of Pharmacology and Clinical Pharmacy, School of Pharmacy, Addis Ababa University, Addis Ababa, Ethiopia

## Abstract

**Background:**

Drug resistance is a universal challenge to malaria control measures. As a result, the development and discovery of new chemotherapeutic agents from medicinal plants having anti-malarial traditional claims are very important. This work, therefore, attempted to evaluate the anti-malarial activity of the aqueous root extract of *E*. *divinorum* using a rodent model of malaria.

**Methods:**

The roots of *E*. *divinorum* were extracted by hot decoction using distilled water. Anti-malarial activity of various doses (100 mg/kg, 200 mg/kg, and 600 mg/kg) of the root aqueous extract was evaluated using the 4-day suppressive test as well as curative and repository tests. Parasitemia, rectal temperature, body weight, PCV, and MST were also determined.

**Results:**

The finding showed that there were a dose-related significant parasitemia chemo-suppression and increment in survival time as compared to the negative control (*p* < 0.001) in all tests. The chemo-suppression effect was higher at 400 mg/kg extract-treated groups in the 4-day suppressive test followed by the curative test. The lowest chemo-prophylaxis effect was observed in 100 mg/kg extract-treated groups in the repository test. Regarding the other parameters, the extract prevented weight loss, temperature drop, and hemolysis in all models but not in a consistent manner.

**Conclusion:**

The current study showed that the aqueous root extract of *E*. *divinorum* possessed a varying degree of anti-malarial activity in all three tests, with greater parasitemia suppression observed in the 4-day suppressive test. The extract produced higher parasitemia chemo-suppression and longer survival time in early infections followed by established and then residual infection.

## 1. Background

The term “Malaria” is derived from the Italian word *mal'aria*, which stands for ‘‘bad air” to describe mainly the swampy areas in Europe, in which the occurrence of the disease was highly prevalent [1]. It is a common disease in tropical and subtropical countries, in which children and pregnant women are high-risk groups [2, 3]. Later on, the protozoan parasite *Plasmodium*, which is the causative agent for the disease, was identified [4]. Moreover, there are more than 100 *Plasmodium* parasite species that can infect many animal species such as reptiles, birds, rodents, monkeys, and humans. From those species, *plasmodium berghei* is a rodent malaria parasite that has a great role in studying the essential cellular and molecular biology of malaria parasites. This is due to its being safe, unable to infect humans, manageability, and exceptional robustness [5, 6]. As a result, the chloroquine-sensitive *P*. *berghei* ANKA strain was an appropriate parasite for the *in vivo* anti-malarial activity of different plant extracts [7].

Most anti-malarial agents, which are available at this time, can be classified according to their mechanism of action and chemical structures. From those different classes of drugs, quinolone derivatives, antifolate combination drugs, antibiotics, and artemisinin derivatives are the most commonly used anti-malarial agents [8]. Even though oral anti-malarial agents are effective to treat uncomplicated malaria, the first-line therapy for uncomplicated *P*. *falciparum* malaria is artemisinin-based combination therapy (ACT) (artemether-lumefantrine) [9]. As a result, ACT (artesunate plus amodiaquine or artemether plus lumefantrine or dihydroartemisinin plus piperaquine) or artesunate (plus clindamycin or doxycycline) or quinine (plus clindamycin or doxycycline) is preferred for the treatment of severe malaria. This is due to the ineffectiveness of a single drug to parasite control associated with the rise in drug resistance [10].

Drug resistance is a global challenge that hinders the malaria elimination strategy, which occurs due to non-adherence, poor drug quality, interactions with other pharmaceuticals, poor absorption, misdiagnosis, and incorrect dosing that lead to treatment failure [11]. So far, the most important cause of anti-malarial drug resistance is gene mutation encoding for transporters, enzymes, and receptors [12, 13]. Besides, drugs with long elimination half-lives are potentially more likely to develop resistance than short-lived (rapidly eliminated) drugs. Hence, the lack of chemical diversity among the anti-malarial drugs in use, which leads to cross-resistance between drugs of the same class of compounds, further aggravates drug resistance [14, 15]. To overcome these problems searching for new anti-malarial agents from medicinal plants have great importance for malaria control measures [16].

*Euclea divinorum* Hiern belongs to the genus *Euclea* and the family Ebenaceae, which is known, by its vernacular name, as Dedeho (Amharic) and Mi'essa (Oromiffa) in Ethiopia is among the commonly used plants for different human ailments [17, 18]. Indeed, studies showed that the crude extract and the solvent fraction of the leaves of *E*. *divinorum* have a renoprotective effect in rats, and the crud extract is endowed with an antimicrobial effect against several bacterial strains and *Candida* maltose [19–21]. Moreover, the hot root decoction is drunk for the treatment of malaria, fever, and anaplasmosis in Kenya and the root of *E*. *divinorum* concocted with water can be taken to treat malaria in a different part of Ethiopia [22–24]. A previous study also showed that methanol root extract of *Euclea divinorum* showed moderate *in vitro* antiplasmodial activity against chloroquine-resistant and sensitive strain of *Plasmodium falciparum* (IC50 = 37.5 *μ*g/ml) as well as low cytotoxicity against MRC-5 (IC50 = 27.5 *μ*g/ml) [25]. However, the anti-malarial activity of this plant is not yet scientifically studied *in vivo*. Thus, this study was initiated to investigate the plants' anti-malarial activity using the traditional claim and an *in vitro* paradigm. Therefore, the aqueous root extract of *Euclea divinorum* was tested *in vivo* using a rodent model of malaria.

## 2. Methods

### 2.1. Plant Material

The fresh root of *Euclea divinorum* was collected from Gedeba Kebele, Raya Kobo Woreda, North Wollo Zone, Amhara Region (North, Ethiopia) in December 2019. Permission was obtained from Raya Kobo Woreda Agricultural office with a license number RK/AO/678/2019. The fresh root was wrapped with plastic sheets during transportation. The specimen of the plant was identified as *Euclea divinorum* Hiern a taxonomist Melaku Wondaferash and a specimen of the plant material was deposited, voucher number (No. FG 001), in the National Herbarium, College of Natural and Computational Sciences, Addis Ababa University for future reference.

### 2.2. Experimental Animals and Parasites

Healthy Swiss albino mice of either sex with weight (20–35 g) or age (6–8 weeks) were used in the study. The mice were obtained from the animal house of the School of Pharmacy, Addis Ababa University. The animals were kept in 12 h light-dark cycle and had free access to a standard pellet laboratory diet and water *ad libitum*. Animals were acclimatized to the laboratory conditions for 1 week before the initiation of the experiment. The care and handling were according to international guidelines for the use and maintenance of experimental animals [26] and the protocol was approved by the School of Pharmacy Ethics Committee with registration number AU/SP/EC/158/19. Chloroquine-sensitive *Plasmodium berghei* ANKA strain was obtained from the Department of Pharmacology, Mekelle University.

### 2.3. Plant Extraction

The root of the plant was thoroughly washed to remove any dead matter or other unwanted particles and then dried under shade at room temperature (25–27°C) with optimal ventilation for 1 month. The dried root plant material was pulverized to a coarse powder using a grinding mill. For 80% methanol extract (ME), 600 gram of air-dried and powdered root of *E*. *divinorum* (100 gram of dried root in 600 ml of 80% methanol) was added in an Erlenmeyer flask and extracted by cold maceration technique, and shacked by using orbital shaker at 120 rpm for 72 h at room temperature. After 72 h, the extract was filtered by Whatman filter paper No. 1. The residue was remacerated for another 72 hours twice and methanol from the combined filtrates was removed under reduced pressure by rotary evaporator at 45 rpm and 40°C to obtain the crude extract. The extract was further concentrated to dryness with a lyophilizer at −40°C and vacuum pressure (200°mBar).

For Aqueous extract (AE), another 400°g of air-dried and powdered root of *E*. *divinorum* (100 mg dried root in 600 ml of distilled water) was extracted by hot decoction for 30 minutes and then cool down to room temperature for 15 minutes. Then, the extract was filtered by Whatman filter paper No. 1. The filtered extract was dried with a lyophilizer at −40°C and vacuum pressure (200 mBar). Finally, 58 gm (9.67%) air-dried powdered 80% methanol root extract of *E*. *divinorum* and about 40 gm (10%) aqueous root extract were obtained. Of note, a pilot study carried out on 80% methanol root extract showed that it is less effective than the aqueous extract, which lead to the use of the latter extract for the study.

### 2.4. Acute Oral Toxicity Testing

An acute oral toxicity study was conducted following OECD 425 guidelines [27]. Accordingly, five mice 6–8 weeks were used and fasted for 4 h before and 2 h after extract administration. After a sighting study single female mouse were weighed and received 2000 mg/kg of the extracts of *E*. *divinorum* orally by oral gavage, and no death was observed within 24 h. Then additional four mice were administered a similar dose of the extract and animals were observed with a 30 min gap after dosing during the first 4 h and for a total of 14 days continuously. At the end of the 14^th^ day, the number of survivors was noted.

### 2.5. Parasite Inoculation

Experimental mice were infected with *P*. *berghei* ANKA strain parasite to induce malaria. The parasites were maintained by intraperitoneal serial passage of blood [28]. *P*. *berghei* infected mice having parasitemia level of 20–30% was used as a donor and the donor mouse was then sacrificed. Blood was collected by a heparinized tube containing 0.5% trisodium citrate. The blood was then diluted with normal saline (0.9%) based on the parasitemia level of the donor mice and the red blood cell (RBC) count of normal mice in 1 ml blood containing 5 × 10^7^ infected RBCs. Then, each mouse was inoculated with 0.2 ml of blood suspension containing 1 × 10^7^*P*. *berghei* parasitized erythrocytes intraperitoneally.

### 2.6. Dosing and Grouping of Animals

The mice were divided into five groups randomly (*n* = 6). Group I (negative control) was treated with distilled water (D/W); group II, III, and IV were treated with 100 mg/kg, 200 mg/kg, and 400 mg/kg of the aqueous extract respectively and group V was treated with the standard drug, chloroquine (25 mg/kg) [20]. The doses for the aqueous extract were chosen after performing a pilot study. For all animals, the oral route of administration was used and the maximum volume administered was 10 ml/1 kg [27].

### 2.7. Determination of Anti-malarial Activity

#### 2.7.1. The 4-Days Suppressive Test

The 4-day suppressive test was employed to test the chemo suppressive activity of the aqueous extract against mice infected with chloroquine-sensitive *P*. *berghei*. Thirty mice were infected on the first day. The mice were then randomly distributed into five groups two-hour after inducing infection and treatment was started immediately as described in the animal grouping and dosing section at day 0. The treatment continued for additional three consecutive days. On the 5^th^ day of the experiment, blood was collected from the tail of each mouse. After that thin smear was prepared on microscope slides to determine the level of parasitemia. Additionally, mice weight, temperature, and packed cell volume (PCV) were measured before infection and at the end of the experiment. To determine the mean survival time (MST) each group of mice was followed for 30 (day 0–day 29) days. Percent parasitemia, percent parasitemia suppression ((%) PS), and MST were calculated using the modified Peters and Robinson formula [29]

(1) $$\begin{array}{c} \%\text{ parasitemia}=\frac{\text{Number of parasitizied RBC}}{\text{Total RBC}}\times 100,\\ \%\text{ suppression}=\frac{\text{Mean parasitemia of negative control}-\text{mean parasitemia of treated group}}{\text{mean parasitemia of negative control}}\times 100,\\ \text{MST}=\frac{\text{Total of days mice survived}}{\text{Total number of mice}}\times 100. \end{array}$$

#### 2.7.2. Curative Test

The curative test was carried out for the aqueous extract to evaluate its curative potential according to the method described in Reyley and Peters [30]. On the first day, mice were injected intraperitoneally with a standard inoculum of 1 × 10^7^*P*. *berghei* infected erythrocytes. After 72 hours, the mice were divided into five groups randomly (*n* = 6) and treated with the respective agents, as described in the grouping and dosing section once daily for 3 days. Thin blood films were prepared from the tail blood of each mouse daily to monitor the levels of parasitemia and the mean survival time for each group was followed for 30 days. Similarly, as described in the 4-day suppressive test section, other parameters were also determined before the 1^st^ dose and on the 7^th^ day of the experiment.

#### 2.7.3. Repository Test

To observe the prophylactic potential of the aqueous extract repository test was done according to the method described by Peters [31]. Thirty mice were randomly distributed into five groups of six mice each and treated as described in the animal grouping and dosing section for 4 days (D1-D4). On the 5^th^ day, all the groups were infected with an inoculum of 1 × 10^7^*P*. *berghei* infected erythrocytes. Blood smears were drawn 72 h postinfection from each mouse to determine parasitemia level. Other parameters were also determined as described in the 4-day suppressive test section pre and posttreatment.

### 2.8. Packed Cell Volume Measurement

The packed cell volume (PCV) was calculated to determine the potential of test extracts to prevent the occurrence of hemolysis in *P*. *berghei* infected mice. Blood was collected from the tail of each mouse in heparinized micro-hematocrit capillary tubes by filling three-quarters of its volume. The tubes were sealed by sealant and placed in a micro-hematocrit centrifuge with the sealed ends outwards. The blood was then centrifuged at 12, 000 rpm for 15 min. The tubes were then taken out of the centrifuge and PCV was determined as follows:

(2) $$\begin{array}{c} \text{PCV}=\frac{\text{Volume of erythrocytes in a given volume of blood}}{\text{total blood volume}}\times 100. \end{array}$$

### 2.9. Determination of Body Weight and Temperature Changes

The body weight of each mouse was measured before infection (day 0) and on day 4 using a sensitive digital weighing balance to observe whether the aqueous extract prevented weight loss for the 4-day suppressive test. In addition, rectal temperature was also measured by a digital thermometer before infection and 4 h after infection, and then daily to check whether the aqueous extract prevents the reduction in rectal temperature. For the curative test, body weight and temperature were measured before infection and from day 3–7 after infection to see the effect of aqueous extract on these parameters.

### 2.10. Preliminary Phytochemical Screening

The aqueous root extract was screened for the presence of different secondary bioactive metabolites using standard tests [32, 33].

### 2.11. Statistical Analysis

The data were expressed as mean ± standard error of the mean. Means of all parameters among groups and within a group were compared using one-way ANOVA followed by Tuckey's post hoc multiple comparison test. In the curative test, two-way ANOVA was also used to analyze the development of parasitemia across days of treatment. *p* values <0.05 were considered statistically significant. SPSS Version 23 Software was used for statistical analysis.

## 3. Results

### 3.1. Determination of Anti-malarial Activity

#### 3.1.1. Acute Oral Toxicity

The acute toxicity test result of this study revealed that the aqueous and 80% methanol extract of *E*. *divinorum* roots was safe by oral route at a dose of 2000 mg/kg. Tested mice showed no significant changes in behaviors and tolerated the administered dose after 72 h of administration. There was no mortality within 14 days of observations and the LD50 value of the extracts was assumed to be greater than 2000 mg/kg in mice.

#### 3.1.2. Pilot Study

The pilot study was conducted to check whether the extract had anti-malarial activity or not and to determine the starting dose of the extracts. In addition, it provides a clue, in which extract a better anti-malarial active ingredient was found from the two extracts to continue for further study using the three anti-malarial models by considering different variables. It showed that the aqueous extract had better chemo suppressive effect as compared to the 80% methanol extract. At 100 mg/kg, 200 mg/kg and 400 mg/kg dose, the percentage suppression was 34.48, 51.53, and 64.8 for aqueous extract and 15.25, 20.69, and 22.14 for 80% methanol extract, respectively (Table 1).

#### 3.1.3. Effect of Aqueous Extract in the Four-Day Suppressive Test

The aqueous root extract of *Euclea divinorum* in the 4-day suppressive test showed a dose-dependent reduction in parasitemia (*p* < 0.001 in all cases) compared to the negative control. The percentage parasitemia suppression of the extract at 100 mg/kg, 200 mg/kg, and 400 mg/kg was 33.49, 47.46, and 62.41, respectively (Table 2). When compared with the positive control (standard drug), the aqueous extract had lower parasitemia suppression at all doses (*p* < 0.001). In addition, the aqueous extract exhibited a significant (*p* < 0.001) increment in survival time at 200 mg/kg and 400 mg/kg as compared to negative control but it was lower than the standard drug. Both parasitemia suppression and survival date were significantly higher at 400 mg/kg compared with the lower dose and middle dose but lower than the positive control.

The extract significantly prevented weight loss at all doses (*p* < 0.001 for 200 mg/kg and 400 mg/kg but *p* < 0.05 for 100 mg/kg) compared to the negative control. There was no statistically significant difference between the 100 mg/kg and 200 mg/kg treated group in preventing weight loss but a significant difference was observed between the higher dose and lower dose treated group (*p* < 0.05) (Table 3). However, the effect of the extract in Attenuation of body weight loss was less (*p* < 0.05) in a lower dose as compared to positive control.

The higher and middle dose extract-treated groups significantly (*p* < 0.01) prevented the reduction in rectal temperature of the infected mice compared to the negative control. The lower dose had no significant detectable difference as compared with the negative control in rectal temperature stabilization but the difference was significant (*p* < 0.01) compared to the positive control (Table 3).

Analysis of the PCV revealed that the higher and middle dose treated groups showed a statistically significant effect (*p* < 0.001) in attenuating PCV decline compared to the negative control. However, the effect in the prevention of PCV reduction was less (*p* < 0.05) in lower dose-treated compared to the higher dose and standard treated groups (Table 3).

#### 3.1.4. Effect of Aqueous Extract in the Curative Test

Throughout the course of treatment, there was a gradual decline in parasitemia level at all doses of the extract and standard drug as compared to the negative control. Only the positive control decreased the parasitemia level to an undetectable level on day 7. In addition, a two-way repeated-measures ANOVA analysis of parasitemia across the course of treatment showed a significant (*p* < 0.001) difference in parasite development. The percentage suppression of the aqueous root extract in Rane's test at 100 mg/kg, 200 mg/kg, and 400 mg/kg was 26.4%, 41%, and 57.79%, (*p* < 0.001), respectively compared to the negative control. However, all test doses had lower (*p* < 0.001) suppression potential compared to the positive control. Besides, there were also significantly different (*p* < 0.001 in all doses) effects in parasitemia suppression when a comparison was made among the different doses of the extract suggesting a dose-dependent suppression effect of the extract (Table 4).

All doses of the extract prolonged the mean survival time significantly (*p* < 0.001) as compared to the negative control but lower than the standard drug. Additionally, there was a significant difference (*p* < 0.05) among different doses of the extract in prolonging survival time (Table 4).

In the curative test, the middle and the higher doses prevented weight loss compared to the negative control (*p* < 0.01) but no significant detectable change was observed in the lower dose. Likewise, there were no significant changes among the test doses as well as between all test doses of the extract positive control in preventing weight reduction.

All doses of the extract significantly attenuated the reduction in rectal temperature (*p* < 0.01 for 200 mg/kg and 400 mg/kg, *p* < 0.05 for 100 mg/kg). However, there was no statistically significant difference among all test doses of the extract as well as between the extract and standard drug in preventing rectal temperature drops.

As shown in Table 5, all test doses of the extract significantly (*p* < 0.001) prevented PCV reduction as compared to the negative control. The extract at a higher dose (400 mg/kg) halted PCV dropping significantly (*p* < 0.05) compared to a lower dose (100 mg/kg). Similarly, the standard drug significantly (*p* < 0.01) prevented PCV decline compared to the lower dose treated group.

#### 3.1.5. Effect of Aqueous Extract in the Repository Test

Although the overall parasitemia suppression effect was much lower than the positive control in the repository test, the entire test dose resulted in a significant chemo suppressive effect (*p* < 0.001 in all doses) in a dose-dependent manner as compared to the negative control.

All treatment groups increased the mean survival time significantly (*p* < 0.001 in all doses) compared to negative control but lower than the standard drug. There were also significant changes in survival time between treated groups as shown in Table 6.

The middle and the higher dose treated group significantly (*p* < 0.01) prevented weight loss as compared to the negative control. Indeed, there was no statistically significant difference observed among these doses and the positive control in preventing weight reduction. However, the prevention of weight loss brought about by the standard was significantly higher (*p* < 0.05) than that of 100 mg/kg of the extract (Table 7).

Only the higher dose treated group attenuated temperature drop significantly (*p* < 0.05) as compared to the negative control. Although the lower and middle doses were unable to prevent the drop in temperature compared to the control, no apparent difference was observed when compared to the higher dose as well as the positive control (Table 7).

As regards PCV, the same pattern observed with that of the rectal temperature was observed except for the higher dose treated group significantly (*p* < 0.05) halted PCV decline as compared to the negative control. The lower dose treated group had statistical significance (*p* < 0.01) and lower potential in preventing PCV reduction compared to positive control. Besides, there was no significantly detectable change between negative control and lower dose as well as middle dose treated groups in preventing PCV reduction.

### 3.2. Phytochemical Screening

Preliminary phytochemical screening revealed the presence of secondary metabolites, including polyphenol, saponins, flavonoids, glycosides, steroids, tannins, and terpenoids (Table 8).

## 4. Discussion

In this study, an *in vivo* rodent malaria model, which accounts for the involvement of the immune system to combat infection and possible prodrug effects that need metabolic reactivation, was employed. The rodent parasite, *P*. *berghei* ANKA was an appropriate parasite for studying the *in vivo* anti-malarial activity of plant extract [7]. Chloroquine was used as the standard treatment drug during this study since *P*. *berghei* is sensitive to it [34]. The most reliable parameters *in vivo* anti-malarial models were percentage parasitemia suppression and survival time [33].

Pilot study in 4-day suppressive test was conducted at three doses to observe the chemo suppressive effect of the extracts (aqueous and 80% methanol extracts). Aqueous extract showed better chemo suppressive effect at 100 mg/kg, 200 mg/kg, and 400 mg/kg than 80% methanol extract at similar doses. This was suggestive of the possible localization of the active components in the aqueous extract. This result is also supported by the folkloric use of the plant *Euclea divinorum*. This may be due to the active components that are responsible for parasite suppression being highly soluble in water than 80% methanol. The result agreed with other studies on *D. angustifolia* that showed the aqueous extract had higher chemo suppressive activity than its hydroalcoholic extract [35].

The aqueous extract of *E*. *divinorum* was investigated for its anti-malarial activity using the 4-day suppressive, curative, and repository tests. These tests were employed to evaluate schizontocidal activity against early infection, curative ability against established infection, and prophylactic activity of the extract against residual infection in *P*. *berghei* infected mice, respectively.

The standard test commonly used for anti-malarial screening of the plant extract is the 4-day suppressive test [31]. In the 4-day suppressive test, the aqueous root extract reduced the level of parasitemia in a dose-dependent manner, suggesting that the plant extract potentially mitigated early malaria infection. The highest parasite suppression (62.41%) was recorded in 400 mg/kg extract-treated groups. The extract prolonged the mean survival time in the three tests but was lower than the standard drug. As a result, the survival time of the standard drug-treated group was higher than the extract-treated group. This may be due to the crude nature of the extract, as it contains a mixture of compounds, compared to the pure compound used as a standard. This finding is in line with the previous report on the leaves' crude extract and solvent fractions of *Olea europaea* [36]. The chemo suppressive potential of the extract was also lower than the standard drug. The middle and higher doses (200 mg/kg and 400 mg/kg) of the aqueous extract-treated groups showed 47.46% and 62.41% chemo-suppression, respectively, as compared to negative control in the 4-day suppressive test. The suppression effect of the extract in the current study is comparable with other reports on the seeds 80% methanolic extract of *Brassica nigra* [37] and the leaves methanolic extract of *Phytolacca dodecandra* [38], which showed 50%, 53.13%, and 50.93%, 55.24% at a dose of 200 mg/kg and 400 mg/kg, respectively.

Although the aqueous root extract had a significant parasitemia suppression in the curative test at all doses, it was lower than the 4-day suppressive test, indicating that the extract had a greater effect in early infection than in established infection, in which the parasite was exponentially growing. This was possibly related to the metabolic process of *E*. *divinorum* extract by mice and the reduction of its concentration in the body that could be associated with the rapid multiplication of the parasite in established infection. This finding is in agreement with another report on *Artemisia turanica* [39], in which the crude extract had greater schizontocidal activity in early infection than the established ones.

Even though all treatment groups in the repository test had considerably reduced parasitemia as compared to the negative control, the percentage of parasitemia suppression and survival time were lower in the repository test than in the other two experiments. The ability of the extract to suppress the parasite in the repository experiment may be hampered by the metabolic inactivation of active components of the extract prior to parasite inoculation [40, 41]. This result is in agreement with another report on the leaves crude extract and solvent fractions of *Olea europaea* [36] and *Morinda lucida* [42], which showed that the extract chemo-suppression and survival time in the repository test, were lower than the curative test and 4-day suppressive test.

The general features of malaria-infected mice are anemia, bodyweight loss, and body temperature reduction [43]. Some of these clinical features are linked with the level of parasitemia directly [44]. The plant extract having anti-malarial activity in traditional claim reports was expected to control some of these manifestations. Ideally, there is a reduction in body weight, temperature, and PCV in *P*. *berghei*-infected mice due to a rapid increase in parasitemia level. The aqueous extract of *E*. *divinorum* prevented weight loss, temperature drop, and PCV decline in *P*. *berghei* infected mice. This finding is in agreement with other studies [45].

Bodyweight loss manifestation of infected mice was due to appetite suppression, disturbed metabolism, and hypoglycemic potential of the parasite. Plant extracts having anti-malarial activity are expected to prevent body weight loss in infected mice associated with the overall pathologic effect caused by the parasite. The mechanism to prevent weight loss might be due to the presence of secondary metabolites in the extract or reduction in the level of parasitemia by the extract [46]. The aqueous root extract of *E*. *divinorum* prevented the weight loss in the higher and middle dose treated group as compared to the negative control in all three tests. This is probably due to a reduction in the level of parasitemia by the extract in infected mice and hence, continues metabolizing and growing without serious hindrances. The result of the present study on body weight is not in agreement with the previous study on other plants [35]. This inconsistency may be due to variation in the concentration of appetite-suppressing components and nutrient contents of the plants.

Ideally, there is a decrease in metabolic rate and rectal temperature in *P*. *berghei* infected mice. The extract having an active compound could ameliorate a decrease in rectal temperature. The temperature stabilization effect of the extract was statistically significant as compared to the negative control in both the 4-day suppressive and curative tests but not in the repository test. This could probably be related to the rapid inactivation of the active component in the extract due to an increment in the level of parasitemia in the repository test. Parasite suppression may indicate that the extract adjusted some pathological processes as well as controlled the immune system of infected mice and offset the decrease in metabolic rate that caused a reduction in internal body temperature. The presence of secondary metabolites such as polyphenol, glycosides, saponins, flavonoids, steroids, and terpenoids that tend to stabilize temperature may contribute to the prevention of parasite-induced temperature decline [35]. This result is in line with other study plants conducted previously [36].

There is a parasite-induced PCV reduction to a hematocrit of 43–44% within 48 h postinfection by rodent malaria [47]. The clearance of uninfected RBCs, erythropoietic suppression, the clearance and/or destruction of infected RBCs, and dyserythropoiesis are the possible mechanisms to cause anemia in both humans and mice [48]. Analysis of PCV in the entire three tests has great importance to evaluate the effectiveness of the extract in preventing hemolysis in *P*. *berghei* infected mice. In the curative and 4-day suppressive test, there was a significant preventive role of the extract to PCV drop at all doses but in the repository test, only the higher dose-treated group significantly prevented PCV reduction as compared to the negative control. The finding showed that the extract could ameliorate anemia by halting parasite-induced RBC destruction. The presence of some secondary bioactive metabolites like tannins and flavonoids having antioxidant activity in the extract may play an important role in preventing RBC from oxidative stress. This is in agreement with other studies [35]. The other possible mechanism might be due to the presence of polyphenol compounds in the extract may prolong the survival of both normal and infected RBCs and prevent the RBCs from oxidative stress. A phenolic compound displays acidic characteristics due to the electron-donating activity of hydroxyl groups, which makes them excellent antioxidants [49]. The protective effect on PCV reduction in this study is concordant with the findings on ethanolic extract of whole fruit of *Lagenaria* [50].

Secondary metabolites could be responsible for the anti-malarial activity of the extract individually or in combination through different proposed mechanisms. Likewise, terpenoids and tannins showed anti-malarial activities by the formation of toxic haem adducts and oxidative damage [51, 52]. The current finding is concordant with other studies [53], which suggest that the presence of tannin and flavonoid can counteract the oxidative damage induced by the malaria parasite due to their antioxidant activity, which prolonged the survival date of infected mice. The other possible mechanism of the plant extract for its anti-malarial activity might be through protein synthesis inhibition, free radical scavenging, and anti-oxidation, DNA intercalation, immunomodulation, or by an unknown mechanism [54]. As a result, the anti-malarial activity of the aqueous root extract of *E*. *divinorum* may be due to the individual or synergistic effect of the aforementioned bioactive secondary metabolites.

The phytochemical screenings showed that the aqueous root extract of *E*. *divinorum* has different secondary bioactive metabolites such as polyphenols, glycosides, flavonoids, terpenoids, steroids, and saponins. The result is in line with previous phytochemical studies done on this plant [55]. There are several secondary bioactive metabolites that have shown anti-malarial activities like terpenes, flavonoids, phenolic compounds, sesquiterpenes, and other related compounds [56, 57]. As a result, those secondary bioactive metabolites have different proposed mechanisms to produce their anti-malarial activity. Regarding that, polyphenols may contribute to the anti-malarial activity by inhibiting haem polymerization and hence resulting in the toxic compound for the parasite [58]. Similarly, steroids were found to exert their antiplasmodial activity by altering the membrane of infected RBCs, which in turn blocks the entrance of essential nutrients into the RBCs and thereby into the parasite [59]. Besides, flavonoids also endeavor their anti-malarial activity through inhibition of the influx of myoinositol and L-glutamine into infected RBCs that are important for the growth of parasites [60], In addition to the aforementioned mechanisms of secondary bioactive metabolites, others may also bring their antiplasmodial activity by directly affecting the pathogen or by indirectly stimulating natural and adaptive defense mechanisms of the host [61].

If the reduction in parasitemia is ≥ 30%, then the agent is considered active in standard screening studies [62]. As the extract meets this criterion, particularly in the middle and higher dose, it can be considered an active agent worthy of further investigation. Depending on percent parasite suppression, an extract can be classified as having good, moderate, or very good *in vivo* anti-malarial activity, if percentage suppression is equal to or greater than 50% at a dose of 500, 250, and 100 mg/kg, respectively [63]. Based on this classification, the aqueous root extract of *E*. *divinorum* exhibited good anti-malarial activity, with a dose-dependent inhibition against *P*. *berghei* infection in mice. This result strengthens the other finding on this plant extract, which showed moderate anti-malarial activity in *in vitro* studies [25]. The current finding is also in agreement with another report on *Brassica nigra* (L.) Koch showed good anti-malarial activity based on the above classification [33].

## 5. Conclusion

The current study showed that aqueous root extract of *E*. *divinorum* possessed a varying degree of anti-malarial activity in all three tests, with greater parasitemia suppression observed in the 4-day suppressive test. The extract produced higher parasitemia chemo-suppression and longer survival time in early infections followed by established and then residual infection. Additionally, the findings also provide evidence to support the *in vitro* study as well as the traditional claims made by the traditional medicine practitioners. Furthermore, investigations should be conducted to isolate and identify the active component and to know the mechanism of action of the plant extract for anti-malarial activity as well as to determine the subacute, subchronic, and chronic toxicities profile of the root extract.

## Figures and Tables

**Table 1:** Parasitemia and chemo suppressive effect on infected mice treated with aqueous extract and 80% methanol extract of root of *Euclea divinorum* in the 4-day suppressive test.

Animal group	Parasitemia level	(%) suppression
CON	34.96 ± 0.62	—
AE100	22.8 ± 0.38	34.48 ± 1.23^*a*3*C*3 *d*3*e*3*f*3*g*3^
ME100	29.2 ± 0.45	15.25 ± 1.32^*a*3 *d*3*f*3*g*1^
AE200	16.7 ± 0.34	51.53 ± 0.98^*a*3*e*3*f*3*g*3^
ME200	27.33 ± 0.79	20.69 ± 1.15^*a*3*f*3*g*3^
AE400	12.13 ± 0.85	64.8 ± 1.24^*a*3*g*3^
ME400	26.83 ± 0.62	22.14 ± 0.88^*a*3^

Data are expressed as mean ± SEM; *n* = 4; a, compared to negative control; b, to 100 mg/kg AE; c, to 100 mg/kg ME; d, to 200 mg/kgAE; e, 200 mg/kg ME; f, 400 mg/kg AE; g, 400 mg/kg ME; 1, *p* < 0.05; 2, *p* < 0.01; 3, *p* < 0.001; CON, control; AE; aqueous extract; ME, 80% methanol extract.

**Table 2:** Parasitemia and survival time of infected mice treated with aqueous root extract of *Euclea divinorum* in the 4-day suppressive test.

Animal group	Parasitemia level	(%) suppression	Survival time
CON	34.58 ± 0.71	—	7.5 ± 0.76
CQ 25 mg/kg	0.00 ± 0.00	100.00 ± 0.00^*a*3^	29.33 ± 0.33^*a*3^
100 mg/kg	23 ± 0.73	33.49 ± 2.03^*a*3*b*3 *d*1*e*3^	9.5 ± 0.76^*b*3 *d*2*e*3^
200 mg/kg	18.17 ± 1.82	47.46 ± 5.044^*a*3*b*3*e*3^	13.33 ± 0.88^*a*3*b*3*e*1^
400 mg/kg	10.33 ± 0.67	62.41 ± 1.85^*a*3*b*3^	17.67 ± 0.677^*a*3*b*3^

Data are expressed as mean ± SEM; *n* = 6; a, compared to negative control; b, to CQ 25 mg/kg; c, to 100 mg/kg; d, to 200 mg/kg; e, to 400 mg/kg; 1, *p* < 0.05; 2, *p* < 0.01; 3, *p* < 0.001; CON, control; CQ, chloroquine.

**Table 3:** Effect of aqueous root extract of *Euclea divinorum* on body weight, temperature and packed cell volume of *P*. *berghei* infected mice in the 4-day suppressive test.

Groups	Body weight			Rectal temperature			Packed cell volume		
	D0	D4	(%) change	D0	D4	(%) change	D0	D4	(%) change
CON	28.54 ± 0.97	26.44 ± 1.15	−7.34	36.67 ± 0.18	35.55 ± 0.21	−3.05	53.29 ± 1.34	50.07 ± 1.37	−6.04
CQ25 mg/kg	29.86 ± 1.04	31.06 ± 0.85	4.02^*a*3^	36.68 ± 0.27	37.12 ± 0.19	1.20^*a*3^	50.83 ± 1.65	50.72 ± 1.59	−0.22^*a*3^
100 mg/kg	28.86 ± 1.76	28.12 ± 1.85	−2.56^*a*1*b*1*e*1^	36.60 ± 0.18	35.97 ± 0.51	−1.72^*b*2^	56.02 ± 3.18	53.86 ± 3.41	−3.86^*b*2*e*1^
200 mg/kg	29.24 ± 2.62	29.58 ± 2.70	1.16^*a*3^	37.03 ± 0.26	37.10 ± 0.25	0.19^*a*2^	58.02 ± 1.63	56.83 ± 1.66	−2.05^*a*3^
400 mg/kg	29.43 ± 1.67	30.65 ± 1.65	4.15^*a*3^	37.08 ± 0.13	37.28 ± 0.87	0.54^*a*2^	53.73 ± 1.07	53.03 ± 1.34	−1.3^*a*3^

Data are expressed as mean ± SEM; *n* = 6; a, compared to negative control; b, to CQ25 mg/kg; c, to 100 mg/kg; d, to 200 mg/kg; e, to 400 mg/kg; 1, *p* < 0.05; 2, *p* < 0.01; 3, *p* < 0.001; CON, control; CQ, chloroquine; D0, pretreatment value on day 0; D4, post- treatment value on day 4.

**Table 4:** Parasitemia, percentage suppression survival time of infected mice treated with aqueous extract of root of *Euclea divinorum* in the curative test.

Groups	D3	D4	D5	D6	D7	(%) suppression	Survival date
CON	20.80 ± 0.56	22.63 ± 0.73	26.09 ± 0.42	33.12 ± 0.33	38.09 ± 0.30	—	8.5 ± 0.22
CQ25 mg/kg	19.37 ± 0.89	11.09 ± 0.75	4.93 ± 0.64	1.32 ± 0.37	0.00 ± 0.00	100 ± 0.00^*a*3^	28 ± 0.58^*a*3^
100 mg/kg	19.12 ± 0.72	22.05 ± 0.77	24.35 ± 0.42	26.26 ± 0.36	28.03 ± 0.36	26.4 ± 0.94^*a*3*b*3 *d*3*e*3^	11.83 ± 0.46^*a*3*b*3 *d*1*e*3^
200 mg/kg	21.32 ± 0.47	21.70 ± 0.40	21.99 ± 0.45	22.28 ± 0.41	22.47 ± 0.52	41 ± 1.36^*a*3*b*3*e*3^	14 ± 0.63^*a*3*b*3*e*3^
400 mg/kg	19.65 ± 0.44	19.40 ± 0.28	19.02 ± 0.25	18.81 ± 0.26	18.36 ± 0.43	51.79 ± 1.13^*a*3*b*3^	16 ± 0.36^*a*3*b*3^

Data are expressed as mean ± SEM; *n* = 6; a, compared to negative control; b, to CQ25 mg/kg; c, to 100 mg/kg; d, to 200 mg/kg; e, to 400 mg/kg; 1, *p* < 0.05; 2, *p* < 0.01; 3, *p* < 0.001; D/W, distilled water; CQ, chloroquine.

**Table 5:** Effect of aqueous root extract of *Euclea divinorum* on body weight, temperature and packed cell volume of *P*. *berghei* infected mice in the curative test.

Groups	Body weight			Rectal temperature			Packed cell volume		
	D3	D7	(%) change	D3	D7	(%) change	D3	D7	(%) change
CON	29.59 ± 1.46	26.50 ± 1.65	−10.44	36.00 ± 0.36	34.92 ± 0.31	−3.00	53.04 ± 1.69	47.99 ± .1.89	−9.52
CQ25 mg/kg	30.18 ± 1.41	29.67 ± 1.55	−1.69^*a*3^	35.43 ± 0.54	35.78 ± 0.25	0.99^*a*2^	51.14 ± 1.62	51.23 ± 1.52	0.18^*a*3^
100 mg/kg	28.17 ± 1.63	26.33 ± 1.66	−6.53	36.67 ± 0.23	36.53 ± 0.19	−0.38^*a*1^	52.71 ± 2.66	50.00 ± 842.5	−3.55^*a*3*b*2*e*1^
200 mg/kg	27.66 ± 0.45	26.58 ± 0.44	−3.90^*a*2^	36.33 ± 0.38	36.53 ± 0.38	0.55^*a*2^	53.99 ± 2.95	53.01 ± 2.94	−1.82^*a*3^
400 mg/kg	30.02 ± 1.40	29.12 ± 1.20	−2.99^*a*2^	36.62 ± 0.28	36.88 ± 0.23	0.71^*a*2^	53.08 ± 3.26	52.67 ± 3.27	−0.77^*a*3^

Data are expressed as mean ± SEM; *n* = 6; a, compared to negative control; b, to CQ25 mg/kg; c, to 100 mg/kg; d, to 200 mg/kg; e, to 400 mg/kg; 1, *p* < 0.05; 2, *p* < 0.01; 3, *p* < 0.001; CON, control; CQ, chloroquine; D3, pretreatment value on day 0; D7, post- treatment value on day 4.

**Table 6:** Parasitemia and survival time of infected mice treated with aqueous root extract of *Euclea divinorum* in the repository test.

Animal group	Parasitemia level	(%) suppression	Survival date
CON	23.01 ± 0.41	—	5.5
CQ 25 mg/kg	6.33 ± 0.23	72.5 ± 1.15^*a*3^	16.17^*a*3^
100 mg/kg	19.78 ± 0.28	18.4 ± 1.74^*a*3*b*3 *d*3*e*3^	8.17^*a*3*b*3 *d*1*e*3^
200 mg/kg	15.14 ± 0.22	31.4 ± 1.52^*a*3*b*3*e*3^	9.67^*a*3*b*3*e*3^
400 mg/kg	12.61 ± 0.43	42.13 ± 1.04^*a*3*b*3^	11.83^*a*3*b*3^

Data are expressed as mean ± SEM; *n* = 6; a, compared to negative control; b, to CQ 25 mg/kg; c, to 100 mg/kg; d, to 200 mg/kg; e, to 400 mg/kg; 1, *p* < 0.05; 2, *p* < 0.01; 3, *p* < 0.001; CON, control; CQ, chloroquine.

**Table 7:** Effect of aqueous root extract of *Euclea divinorum* on body weight, temperature and packed cell volume of *P*. *berghei* infected mice in the repository test.

Groups	Body weight			Rectal temperature			Packed cell volume		
	D0	D3	(%) change	D0	D3	(%) change	D0	D3	(%) change
CON	24.96 ± 0.83	23.31 ± 1.20	−6.61	35.6 ± 0.41	34.67 ± 0.43	−2.61	54.45 ± 2.04	52.87 ± 1.98	−2.9
CQ25 mg/kg	24.34 ± 1.12	24.8 ± 1.21	1.89^*a*3^	35.7 ± 0.40	35.87 ± 0.25	0.47^*a*2^	55.84 ± 3.35	56.45 ± 3.10	1.09^*a*2^
100 mg/kg	24.20 ± 0.88	23.30 ± 0.76	−3.72^*b*1^	36.0 ± 0.35	35.42 ± 0.19	−1.16	55.14 ± 0.86	54.03 ± 1.10	−2.01^*b*2^
200 mg/kg	29.37 ± 1.07	29.35 ± 1.27	−0.07^*a*2^	36.03 ± 0.29	35.67 ± 0.28	−1.00	51.69 ± 2.10	51.28 ± 2.01	−0.79
400 mg/kg	27.17 ± 1.13	27.32 ± 1.12	0.56^*a*2^	36.17 ± 0.39	36.13 ± 0.47	−0.11^*a*1^	54.24 ± 1.02	54.13 ± 0.87	−0.20^*a*1^

Data are expressed as mean ± SEM; *n* = 6; a, compared to negative control; b, to CQ25 mg/kg; c, to 100 mg/kg; d, to 200 mg/kg; e, to 400 mg/kg; 1, *p* < 0.05; 2, *p* < 0.01; 3, *p* < 0.001; CON, control; CQ, chloroquine.

**Table 8:** Preliminary phytochemical screening of the aqueous root extract of *Euclea divinorum*.

Metabolites	Aqueous root extract
Polyphenol	+
Flavonoids	+
Glycosides	+
Steroids	+
Alkaloids	−
Tannins	+
Terpenoids	+
Saponins	+

+ = present, − = not detected.

## Data Availability

All data supporting the results are available on the hand corresponding authors upon request.

## Acronyms

MST: Mean survival time
PCV: Packed cell volume
RBC: Red blood cell
SEM: Standard error of the mean
WHO: World health organization.
